# Food co-consumption network as a new approach to dietary pattern in non-alcoholic fatty liver disease

**DOI:** 10.1038/s41598-023-47752-y

**Published:** 2023-11-24

**Authors:** Mohammad Mehdi Naghizadeh, Saeed Osati, Reza Homayounfar, Ali Masoudi-Nejad

**Affiliations:** 1 Laboratory of Systems Biology and Bioinformatics (LBB), Institute of Biochemistry and Biophysics, University of Tehran, Tehran, Iran; 2 Noncommunicable Diseases Research Center, Fasa University of Medical Science, Fasa, Iran; 3 National Nutrition and Food Technology Research Institute, Faculty of Nutrition Sciences and Food Technology, Shahid Beheshti University of Medical Sciences, Tehran, Iran

**Keywords:** Metabolic disorders, Nutrition disorders, Liver diseases, Nutrition disorders

## Abstract

Dietary patterns strongly correlate with non-alcoholic fatty liver disease (NAFLD), which is a leading cause of chronic liver disease in developed societies. In this study, we introduce a new definition, the co-consumption network (CCN), which depicts the common consumption patterns of food groups through network analysis. We then examine the relationship between dietary patterns and NAFLD by analyzing this network. We selected 1500 individuals living in Tehran, Iran, cross-sectionally. They completed a food frequency questionnaire and underwent scanning via the FibroScan for liver stiffness, using the CAP score. The food items were categorized into 40 food groups. We reconstructed the CCN using the Spearman correlation-based connection. We then created healthy and unhealthy clusters using the label propagation algorithm. Participants were assigned to two clusters using the hypergeometric distribution. Finally, we classified participants into two healthy NAFLD networks, and reconstructed the gender and disease differential CCNs. We found that the sweet food group was the hub of the proposed CCN, with the largest cliques of size 5 associated with the unhealthy cluster. The unhealthy module members had a significantly higher CAP score (253.7 ± 47.8) compared to the healthy module members (218.0 ± 46.4) (*P* < 0.001). The disease differential CCN showed that in the case of NAFLD, processed meat had been co-consumed with mayonnaise and soft drinks, in contrast to the healthy participants, who had co-consumed fruits with green leafy and yellow vegetables. The CCN is a powerful method for presenting food groups, their consumption quantity, and their interactions efficiently. Moreover, it facilitates the examination of the relationship between dietary patterns and NAFLD.

## Introduction

Non-Alcoholic fatty liver disease (NAFLD), which is the result of the accumulation of fat in liver tissue^1^, is one of the leading causes of chronic liver disease in developed societies. A meta‐analysis study showed that the prevalence of NAFLD among the adult population from all over the world is about 25%; it also showed that the prevalence of this disease in the Middle East is about 32%—that was, a more significant percentage of cases is suffering from this medical condition in this part of the world^2^. Obesity, Type 2 diabetes, dyslipidemia, and metabolic syndrome are the approved risk factors in NAFLD. Metabolic syndrome is considered to be among the factors that are of particular concern to researchers because NAFLD is regarded as the most important hepatic manifestation of this syndrome^3^. According to many recent studies, dietary patterns are a risk factor for NAFLD^4,5^.

From the different types of dietary patterns, the rich fat diets may lead to fatty liver diseases^6^. Diets containing a large amount of sugar may also contribute to early stages of NAFLD in children^7^. As evidenced by some studies, there is an association between carbohydrate-based diets and NAFLD^8^. An increase in the consumption of vegetables, legumes, and fruits is associated with a likely reduction in the development of this disease^9^. Some researchers have merely focused on the effect of dietary patterns, in contrast to others, who have regarded various food groups as risk factors in NAFLD. For instance, the odds of NAFLD decreased in the Lebanese traditional diet^10^ or following healthy^11^ dietary patterns. Also, based on some other studies, Mediterranean dietary patterns, for example, decrease the likeliness of NAFLD^12^.

What is a dietary pattern? A dietary pattern has been made up of three main components. It is defined as a variety, quantity, and combination of different food items and beverages in a diet^13^. Conventional studies have often focused on the variety and quantity components without taking into consideration the combination. For example, in contrast to many valuable reports on the quantity of tea and coffee consumption^14^, only a few reports have been given on the co-consumption of tea and coffee, which without providing us with more information about their co-consumption, just give some information about the percentage of people who consume both tea and coffee^15^. Furthermore, in the case of the other food groups, few studies have focused on their co-consumption; and none of the studies has examined the co-consumption of food items as a risk factor for diseases, especially in NAFLD.

Iqbal et al.^16^ are the first ones who employed the network representation method to describe the dietary patterns. They used their proposed network, named the intake network, to analyze the dietary patterns of the adult population living in Germany and concluded that meat played a major role in their dietary patterns. They did not extend their definition to the disease-related intake network. In the present study, co-consumption has been defined as a pairwise correlation between the quantity of two food items eaten together. A method, entitled the co-consumption network (CCN), has been introduced to measure the co-consumption of two food groups. This network has been detailed through various previously developed network analysis methods in the field of social science^17^. A typical network should represent the food elements as well as the quantity of their consumption, and the relationship between food groups and their strength in one shot. It should also depict the dietary patterns.

Although dietary patterns strongly correlate with NAFLD^18^, their mechanism in the development of NAFLD is still not fully understood^7^. In this study, we first examine the common consumption of food groups in dietary patterns through a new definition named the co-consumption network (CCN). Then, we discuss the relationship between dietary patterns and NAFLD based on our network. This study also aims to introduce the co-consumption term as well as the co-consumption network analysis in nutrition.

## Results

### Co-consumption network

**Figure 1:**
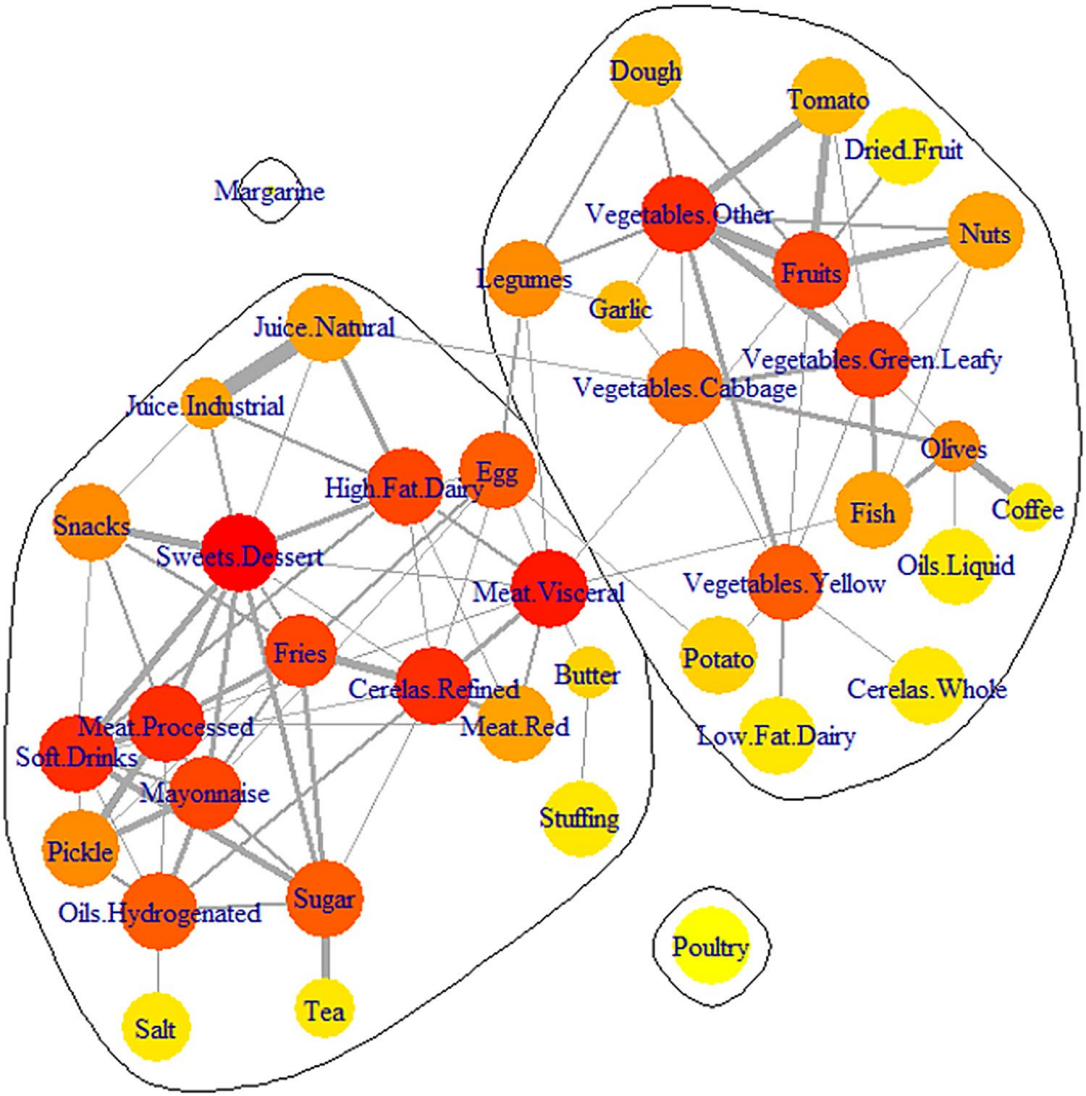
Co-consumption network (CCN). In this network, the nodes are food groups, the edges are spearman correlation coefficients > 0.2, edge thickness is the correlation coefficient, node size is weighted by their consumption, the nodes are colored by degree (red is higher degree and yellow is lower degree). The CCN has two isolated nodes (margarine and poultry) and also two clusters.

In this study, 1500 individuals (570 males and 930 females) aged 40.9 ± 11.6 years, with the BMI of 27.9 ± 4.7 kg/m^2^ participated. The consumed foods were categorized into 40 groups with correlation coefficients ranging from − 0.383 to 0.534 and an upper quartile equal to 0.135 (Supplementary Fig. S2). There were 96 connections (edges) between 40 nodes, provided that the significant and positive correlation coefficients which were greater than 0.2, were picked as a soft threshold. In this case, each edge located between the two nodes was demonstrating the similar consumption patterns of these two food groups observed in the participants. For example, sweet dessert and soft drink had the same pattern of consumption and were therefore labeled “co-consumed”. The introduced network, named the co-consumption network (CCN), was presented in Fig. 1. The CCN was developed to show the dietary patterns of the participants. This network had a main connected component with two isolated nodes, margarine and poultry, that did not have the same pattern of consumption as the other ones.

The edge thickness in the CCN was chosen as the correlation coefficient. The thicker one was a food group with a high correlation. Natural and industrial juice groups had the highest correlation (r = 0535). These two groups were remarkably similar in their consumption pattern. Binarization generated the consumption score of the food groups. This score was added to the network as a node size. Bigger nodes were the highly consumed food groups. The seven food groups low-fat dairy, fruits, tomato, other vegetables, snacks, nuts, and hydrogenated oils (n = 1125) had the highest and margarine (n = 136) had the lowest level of consumption (Table 1).

**Table 1:** Food groups consumption and centrality indices of them.

Food groups	Mean	Standard deviation	Median	Consumption	Modules	Degree	Betweenness centrality	Closeness centrality
Butter	4.3	7.3	0.6	748	Unhealthy	2	0.054	0.352
Cereals.Refined	379.8	254.0	328.5	1124	Unhealthy	9	0.082	0.440
Cereals.Whole	105.0	117.6	70.9	1124	Healthy	1	0.000	0.280
Coffee	23.1	104.5	0.0	699	Healthy	1	0.000	0.262
Dough	170.6	214.5	98.0	1059	Healthy	3	0.001	0.339
Dried.Fruit	22.9	268.8	2.3	1119	Healthy	1	0.000	0.314
Egg	23.6	17.7	21.4	1123	Unhealthy	8	0.112	0.457
Fish	14.7	31.4	7.3	1121	Healthy	4	0.097	0.411
Fries	14.1	42.8	4.0	1047	Unhealthy	8	0.023	0.381
Fruits	912.3	1823.9	712.3	1125	Healthy	8	0.175	0.451
Garlic	1.1	4.0	0.0	736	Healthy	3	0.003	0.352
High.Fat.Dairy	114.5	195.6	36.0	1123	Unhealthy	8	0.040	0.430
Juice.Industrial	40.1	77.0	8.0	748	Unhealthy	4	0.005	0.359
Juice.Natural	61.7	92.9	20.4	1113	Unhealthy	4	0.068	0.407
Legumes	70.9	83.9	52.3	1124	Healthy	5	0.068	0.416
Low.Fat.Dairy	373.9	330.8	306.6	1125	Healthy	1	0.000	0.280
Margarine	0.7	3.2	0.0	136	Isolated	0	0.000	0.000
Mayonnaise	4.5	7.1	2.0	1070	Unhealthy	8	0.027	0.385
Meat.Processed	7.7	12.5	3.2	1122	Unhealthy	9	0.084	0.430
Meat.Red	56.3	42.8	45.9	1124	Unhealthy	5	0.001	0.411
Meat.Visceral	3.7	7.6	1.6	1122	Unhealthy	10	0.376	0.521
Nuts	14.3	41.2	6.5	1125	Healthy	4	0.003	0.346
Oils.Hydrogenated	25.1	35.4	15.0	1125	Unhealthy	7	0.056	0.343
Oils.Liquid	12.8	25.0	6.0	1108	Healthy	1	0.000	0.262
Olives	22.0	371.4	0.4	745	Healthy	5	0.109	0.352
Pickle	63.4	361.7	29.1	1124	Unhealthy	5	0.001	0.333
Potato	23.7	33.5	15.3	1123	Healthy	2	0.035	0.374
Poultry	24.4	25.8	14.9	1124	Isolated	0	0.000	0.000
Salt	15.3	15.7	9.0	995	Unhealthy	1	0.000	0.257
Snacks	24.5	37.8	8.7	1125	Unhealthy	5	0.003	0.339
Soft.Drinks	61.6	126.9	16.3	1113	Unhealthy	9	0.017	0.359
Stuffing	4.5	8.0	1.2	1107	Unhealthy	1	0.000	0.262
Sugar	28.9	29.7	21.7	1124	Unhealthy	7	0.057	0.352
Sweets.Dessert	13.6	18.5	6.2	1124	Unhealthy	11	0.111	0.446
Tea	824.4	659.9	735.0	873	Unhealthy	1	0.000	0.262
Tomato	229.1	193.5	176.7	1125	Healthy	3	0.000	0.343
Vegetables.Cabbage	6.0	16.4	1.0	1124	Healthy	6	0.089	0.398
Vegetables.Green.Leafy	58.9	544.2	23.0	1124	Healthy	8	0.053	0.385
Vegetables.Other	288.5	202.2	231.9	1125	Healthy	9	0.050	0.394
Vegetables.Yellow	24.3	35.4	12.2	1098	Healthy	7	0.128	0.385

The unit of mean, standard deviation and median are g/day. The consumption columns are number of participants that labeled as consumer in binarized dataset (supplementary section III).

The number of edges connected to a node was regarded as its degree. The node with the highest degree is the hub of the network. In the CCN, the food group which was co-consumed with many food groups, was determined by the highest node degree. The sweet dessert group (11°) was the hub of the CCN and it was co-consumed with more food groups. In Fig. 1, the node degrees were distinguished by color. The red nodes were the hubs and the yellow ones were the food groups with lower degrees.

The CCN had a clique with size 5. A clique is a subnetwork, whose every two nodes are connected. This clique contained red meat, visceral meat, eggs, high-fat dairy, and refined cereals. All of these five food groups had the same consumption patterns and therefore were co-consumed.

The modularity score of the CCN was 0.418. The score had a range of − 1 to 1, and the positive value indicated that the network had a modular structure. So, network clustering was done using the label propagation algorithm. This algorithm, which is a topological-based one, clustered the network without considering the consuming matrix. It divided the main connected component of the CCN into two modules. The module is a subnetwork with more intrinsic and less extrinsic connectivity.

Salt, oils, meats, sweets, industrial drinks, and some others (20 nodes) fell into a bigger cluster. Vegetables, fruits, legumes, and several others (18 nodes) fitted into another cluster. We named the first group the unhealthy and the second the healthy clusters. The unhealthy module had 20 nodes and 57 edges and the healthy one 18 nodes and 33 edges. The unhealthy module had more density (connection per node) than the healthy one (2.9 and 1.8, respectively). The results showed that in the unhealthy module, the co-consumption of the food groups happened more than the healthy one.

Betweenness centrality with the range 0–1 is a measure that a food group lies on paths between others for this reason this node may have considerable influence within the network. The betweenness centrality of the visceral meat food group was 0.375, with the highest one, it placed in the largest clique and had a position between two modules (Table 2). Closeness centrality with the ranges from 0 to 1 indicates that a food group is close to others in the network. The highest closeness centrality in the CCN was 0.457 for the egg.

**Table 2:** Comparison of characteristics between unhealthy and healthy modules consumers.

		Unhealthy (n = 323)		Healthy (n = 326)		*P*-value
		Mean	SD	Mean	SD	
Age (years)		44.9	11.2	35.3	10.8	< 0.001
Energy (Kcal/d)		2605.1	437	2414.0	429.9	< 0.001
BMI (kg/m^2^)		29.5	4.8	25.6	4.6	< 0.001
CAP score (Db/m)		253.7	47.8	218.0	46.4	< 0.001
		n	%	n	%	
Sex (Female ratio)		304	94.1	77	23.6	< 0.001
Steatosis grade	S0	114	35.3	219	67.2	< 0.001
	S1	53	16.4	37	11.3	
	S2	65	20.1	45	13.8	
	S3	91	28.2	25	7.7	

### Gender-related differential co-consumption network

**Figure 2:**
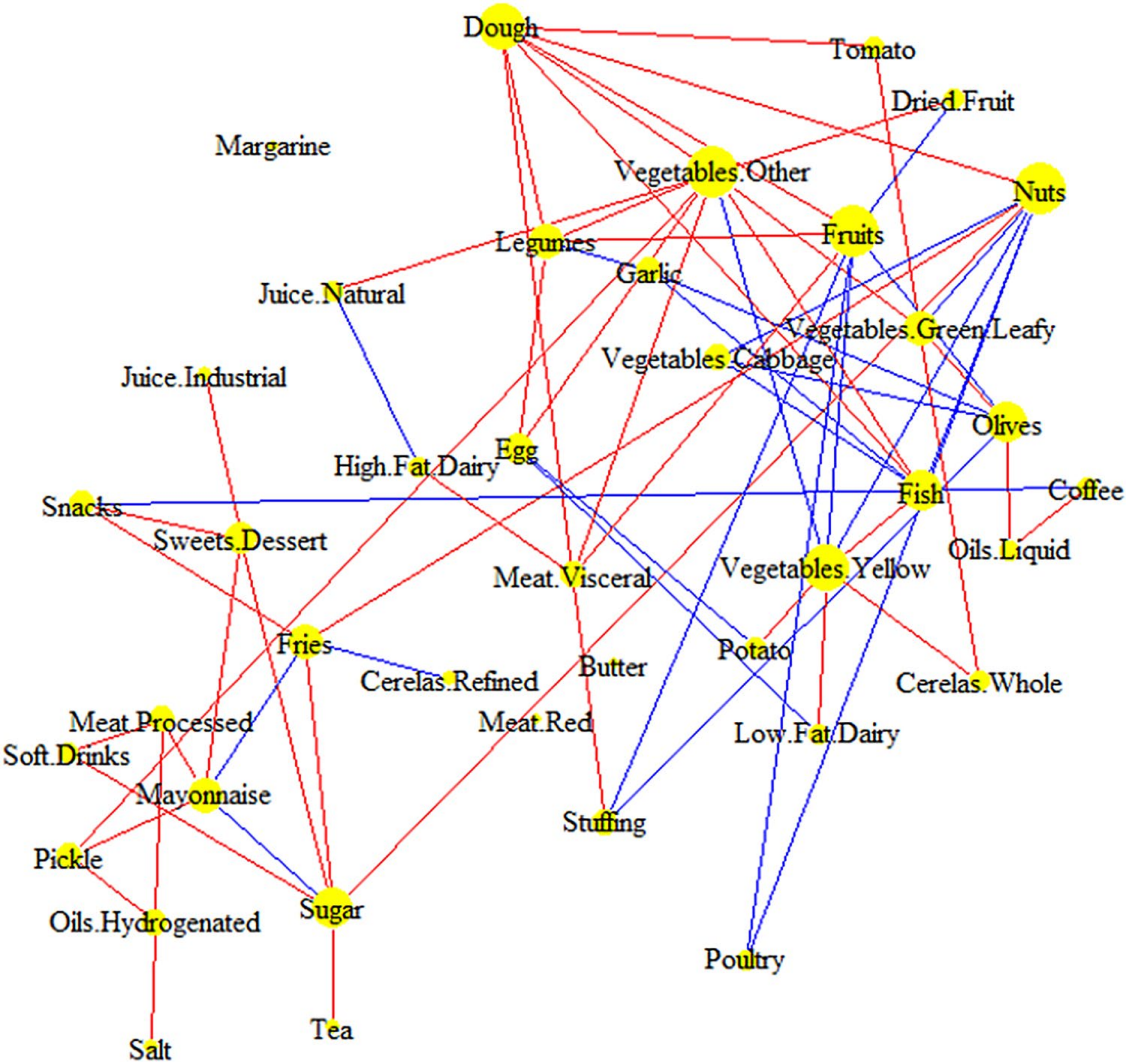
Gender dependent differential co-consumption network. In this network, the edges are chosen if they co-consumed only in males (blue) or females (red). The node size is weighted by degree.

We divided the data by gender and constructed two separate male and female CCNs (Fig. S1). The gender-related differential co-consumption network was reconstructed by the edges which had appeared only in one of the male or female networks (Fig. 1). The gender-related differential co-consumption network was reconstructed by the edges which had appeared only in one of the male or female networks (Fig. 2). The edges of this network were the co-consumption patterns of the food groups that occurred in only one of male (blue) or female (red) networks. The differential CCN helped distinguish between the genders’ exclusive patterns of co-consumption. For instance, males were consuming soft drinks independently but were co-consuming the egg with potato and low-fat dairy. Vice versa, the females were consuming the egg independently but were co-consuming soft drinks with processed meats and sugar.

### Age trend network

**Figure 3:**
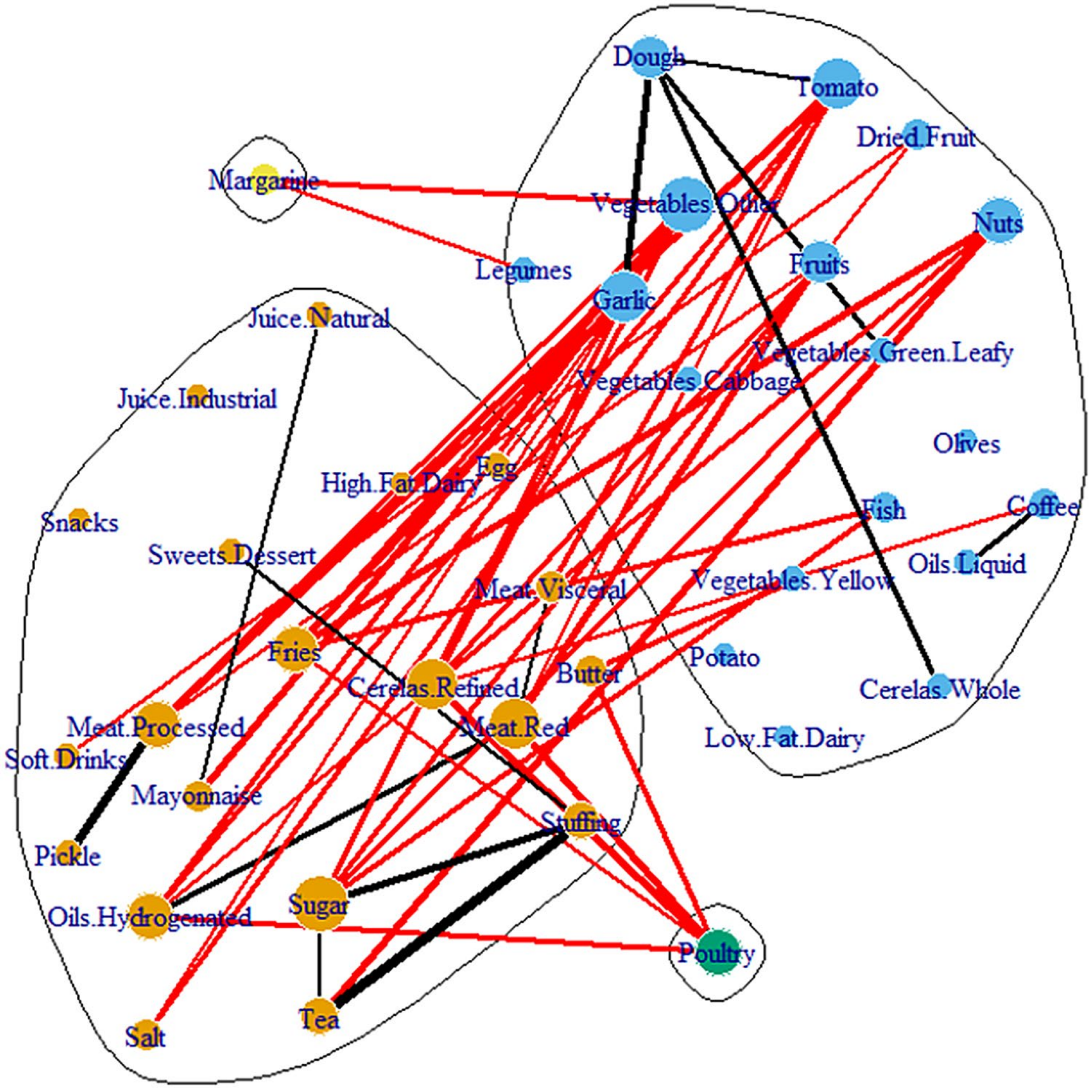
Age trend co-consumption network. In this network, the edges are chosen if they have a significant, positive and increasing correlation during three age categories. The black edges represent a within and the red edges, between modules co-consumption. Edge thickness was defined based on the increase in correlation. The node size is weighted by degree.

The age trend network was introduced to distinguish between the different co-consumption patterns of three distinct age groups. The edges of this network illustrated the co-consumption patterns which were happening at a continually increasing rate as people were getting older. For instance, it was shown that pickles and processed meats as two food groups have been more co-consumed by the elderly. In this network, the black edges were represented within the module's co-consumption and the red ones between the module's co-consumption. The edge thickness was defined based on an increase in the correlation. As shown in Fig. 3 this network was full of between the module edges.

### Comparison of two modules members

Module membership detection showed that 323 participants (21.5%) belonged to the unhealthy, 326 (21.7%) belonged to the healthy module, and 821 participants (56.8%) belonged to both or none of the modules. As presented in Table 2, the female ratio in the unhealthy module was higher (*P* < 0.001). The unhealthy module members had also significantly higher ages (*P* < 0.001), higher BMI (*P* < 0.001), and a higher intake of energy (*P* < 0.001). Finally, the CAP score of the unhealthy module members was 253.7 ± 47.8, which was significantly higher than the members of the healthy module 218.0 ± 46.4 (*P* < 0.001). Therefore, 91 (28.2%) participants in the unhealthy module and 25 (7.7%) in the healthy module had fatty liver grade S4 (*P* < 0.001).

### Module-related cliques

The module-related cliques were presented in Table 3. There was one clique with size 5. This clique’s food groups (red meat, visceral meat, eggs, high-fat dairy, and refined cereals) had been consumed by 62% of the unhealthy module members (201 persons) and 13.8% of the healthy module members (27 persons) (*P* < 0.001), therefore, it was associated with the unhealthy module members. According to the results, the food groups such as meats, soft drinks, and sweets were the key elements of the unhealthy module, while vegetables and fruits were the key elements of the healthy module (Table 3).

**Table 3:** The maximal size of unhealthy/healthy modules related to cliques with the comparison of consumption in the module consumers.

Clique size	Clique food groups	Module	Unhealthy consumer (n = 323)	Healthy consumer (n = 326)	*P*-value
5	Red meat/visceral meat/egg/high-fat dairy/refined cereals	Unhealthy	201 (62.2%)	27 (13.8%)	< 0.001
4	Refined cereals/sweets and dessert/sugar/soft drinks	Unhealthy	261 (80.8%)	4 (1.2%)	< 0.001
4	Processed meat/mayonnaise/hydrogenated oils/soft drinks	Unhealthy	233 (72.1%)	27 (8.3%)	< 0.001
4	Fruits/yellow vegetables/green leafy vegetables/other vegetables	Healthy	51 (15.8%)	259 (79.4%)	< 0.001
4	Cabbage vegetables/yellow vegetables/green leafy vegetables/other vegetables	Healthy	42 (13.0%)	232 (71.2%)	< 0.001
4	Fruits/green leafy vegetables/other vegetables/nuts	Healthy	63 (19.5%)	245 (75.2%)	< 0.001

### Diseases Differential co-consumption network

**Figure 4:**
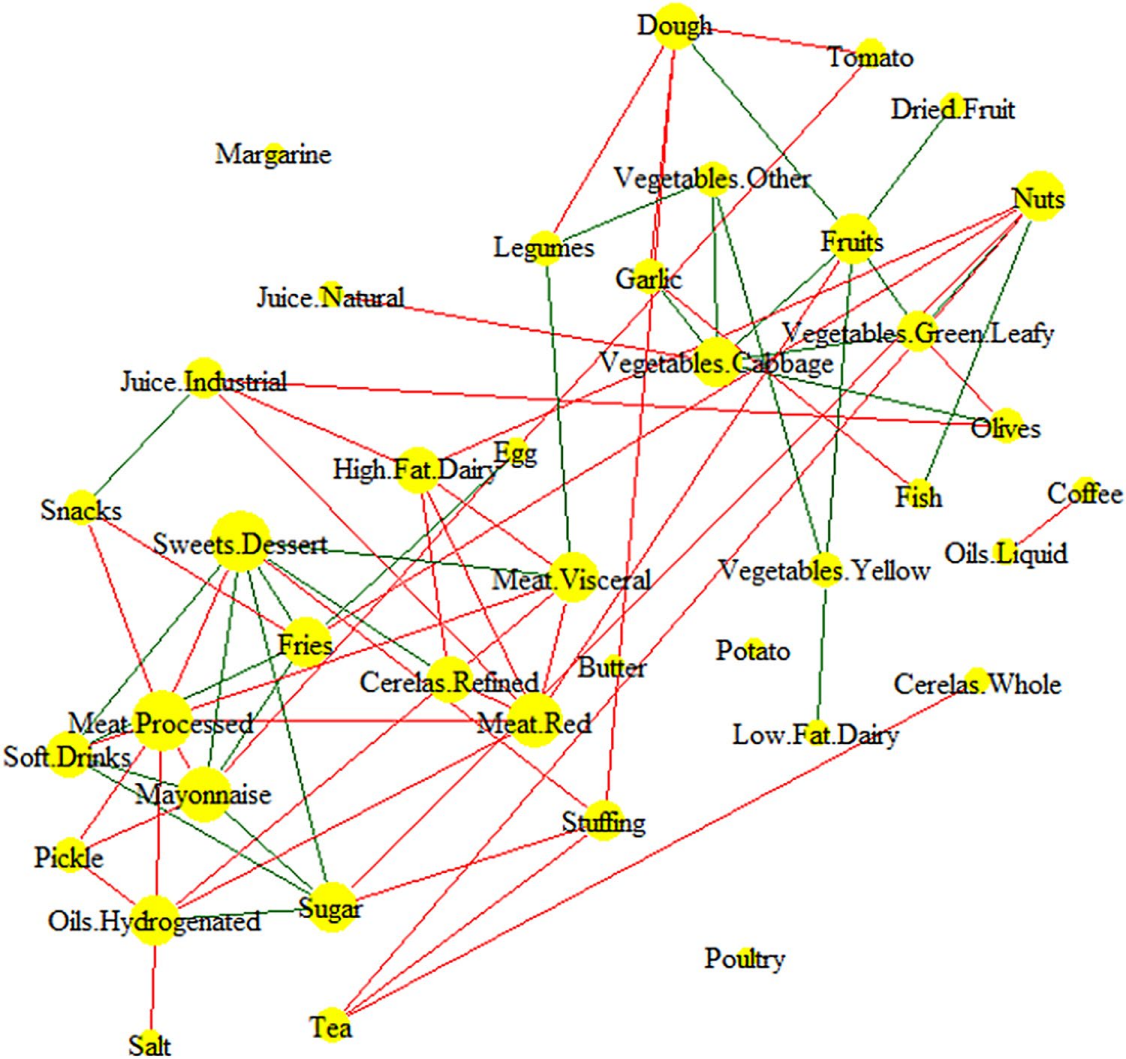
NAFLD dependent differential co-consumption network. In this network, the edges are chosen if they co-consumed only in normal liver (green) or NAFLD (red). The node size is weighted by degree.

In this study, 47.7% of the participants (715 persons) had a CAP score lower than 238, which was indicating that they had a normal liver, and 19.4% of the participants (291 persons) had a CAP score higher than 293, showing that they were patients with a fatty liver. After dividing the data by disease, two separate NAFLD and normal CCNs were constructed in the same manner (figure S2). The fatty liver differential CCN was one of the useful methods that helped facilitate drawing a distinction between the different co-consumption patterns of healthy people with normal liver and patients with fatty liver. Based on the results produced by this network, in the case of the patients with NAFLD, processed meat had been co-consumed with red and visceral meat also, with mayonnaise and soft drinks. Moreover, sugar, stuffing, and sweet food groups made another co-consumption triangle. While in the healthy participants’ case, the fruits group had been co-consumed with the green and other vegetable groups (Fig. 4).

## Discussion

Introducing the dietary patterns as a network (CCN) was the first aim of the present study and was achieved in Fig. 1. The CCN facilitated presenting the food groups and the quantity of their consumption as well as the co-consumption of food groups and the intensity of their interaction in one shot. Through this network, the nutritionists found out the healthy and unhealthy food clusters and the hub of the CCN (sweets) as well as the intra- (soft drinks and processed meat) and extra- (fruits and visceral meat) module interactions, which in turn will lead to setting an exact and early dietary intervention for the clients. The connection between the two food groups in the CCN was interpreted as a correlation between their consumption. For instance, there was an edge between soft drinks and processed meat. So, a reduction in one food group could reasonably result in a decrease in the other one; and encouraging participants to control one of the food groups’ consumption could lead to bringing the other one’s consumption under control.

The network representation of dietary patterns as the intake network was previously suggested by Iqbal et al.^16^. They employed the Gaussian graphical model to predict the connection between the food groups^19^. They first constructed two separate networks for males and females and then made a comparison between them. The result of their introduced approach was a sparse network. Although the network sparsity made it a simple interpretation of the dietary patterns, extending it to the diseases did not prove its interpretive ability. They used a soft threshold besides a nonparametric correlation to make the connections. This way, they developed a denser network that could present the dietary patterns better. They also employed the Bayesian inference^20^ and LASSO regression techniques^21,22^. In CCN, the sweet food group was the hub of the network and their consumption was correlated with many other food groups’ consumption patterns. Also, the visceral meat group and the egg were another two important food groups with the highest betweenness and closeness centrality. These characteristics make potential them to be the focal point of the nutritional intervention. This food group was also the focal point of some clinical trials to prevent or control obesity in children^23^. Sweet-bread spreads, cakes and cookies, desserts, confectionery, and canned foods were an isolated part of the Iqbal et al.^16^ intake network. However, there were many reports about the harmful effects of salt^24^, particularly considering the metabolic diseases^25^. In the CCN, salt was an isolated node with just one connection to solid oils. In comparison to the sweet-based intervention which could affect many connected food groups, the salt-based intervention could affect only salt and solid oils.

In the present study, the CCN was partitioned into two clusters, named the healthy and unhealthy modules. These two clusters were similar to the previously reported patterns except for some small differences^26^. In this research, nuts fell into the healthy cluster that was similar to the healthy/western patterns followed by the Swedish wome^27^ as well as the Mediterranean/western patterns found in the Spanish SUN project^28^. The unhealthy module was denser than the healthy one, which meant that there was a high correlation between the food group consumption in the unhealthy module. Two cliques of the CCN fell into the unhealthy module. An effective intervention to transition from unhealthy to healthy dietary patterns indicated that there must be close attention to the linkage between the two modules.

Introducing the gender-specific CCN was among the other findings of the present research. The male and female CCNs, along with the differential CCN will facilitate the designing of an effective treatment for males and females by the nutritional interventionists. For example, since in the case of males, soft drinks were an isolated node, the intervention on it had to be designed independently. In females’ case, processed meat was the focal point, because this food group connected to soft drinks, which could lead to a reduction in soft drinks. There were some reports on the relationship between soft drinks and fast foods in females^29^. There were also some reports on a need for gender-differentiated intervention programs for soft drinks^30^.

In this study, the age trend network was full of between-module connections. This meant that the number of the elderly who were consuming both the healthy and unhealthy food groups exceeded the number of young people. In contrast, young people were following just one of the dietary patterns (healthy or unhealthy) and consuming only one of the modules’ food groups. Such a shift in dietary patterns from healthy to unhealthy was reported in the USA^31^ and Italy^32^. The existing bridge between the two dietary patterns could help people to make the transition from the unhealthy to the healthy modules during the correctly designed intervention. A lack of such a connection could make the transition process difficult as in young people’s case in the present study.

Assigning participants to the modules showed that the unhealthy food group’s consumers had a high intake of energy and a higher BMI. There were many reports about the effects of the western or unhealthy dietary patterns on energy intake^33^ and BMI^34,35^. The present analysis showed that there exists a direct relationship between unhealthy dietary patterns and the fatty liver index. Recently, the effect of dietary patterns on liver fibrosis in NAFLD has been studied^36^. There were also some reports on improvements in the dietary patterns and how to shift from the unhealthy to the healthy dietary patterns as well as some accounts of the patients suffering from NAFLD and how the hepatic steatosis and steatohepatitis helped develop a fatty liver^37,38^. Studies showed that the Mediterranean diet intervention could help control the hepatic steatosis^39^. According to our previous study, the mechanism of the effects of the dietary patterns on the accumulation of fat in the liver may be mediated by the energy intake and obesity^40^.

The findings of the previous studies indicated that the intake of vegetables^11,18,41–43^, fruits^10,11,18,41^ and high-fibred diets^43^, milk^18^, coffee^44–46^, fish^11,41^, potatoes^18^, grains^11^, and legumes^11^ had good protection against NAFLD. In contrast, the intake of red^18,42,47^, visceral^18^ and processed^47^ meat, grains^43,48^, sweets^18^, soft drinks^42,49,50^ and sugar-rich diets^7,51^, oils^18^, and fatty diets^52^ can significantly help increase the risk of NAFLD. In the present study, in addition to the consumption of food groups, their co-consumption was also presented as the network cliques. Moreover, two unhealthy module-related cliques with size 5 and five healthy module-related cliques with size 4 were discovered. These cliques and the FLI-differentiated CCN can serve as a template for designing an accurate nutritional intervention.

## Conclusion

In conclusion, the CCN was a powerful method that helped facilitate presenting the food groups, the quantity of their consumption, and their interaction in one shot. This network and its delivered results highlighted the co-consumption of food groups as a lost concept in the studies associated with nutrition. The CCN was also employed to extract various dietary patterns and discover the existing differences in the dietary patterns of the subpopulations. In the present study, a new approach was used to apply the co-consumption network (CCN) to find the relationship between dietary patterns and NAFLD. Moreover, introducing a template for designing an accurate dietary intervention was one of the other applications of the intake network reconstruction. This approach can be extended by changing the network parameters such as applying the negative correlations beside the positive ones.

This study provided a more comprehensive understanding of the effects of dietary co-consumption in NAFLD patients compare to normal liver participants that could be useful for future clinical implications. Since meat dietary pattern induced a higher NAFLD risk^53^ and dietary fatty acids establish a crucial bridge between diet and NAFLD^54^. In our study, the processed, red, visceral meat also sugar, stuffing, sweet were two cores of differential CNN in the NAFLD patents they could be as a core of the clinical dietary intervention.

## Materials and methods

### Participants

One thousand five hundred individuals were selected using a convenience sampling method among persons visiting a nutrition counseling center in Tehran from April 1, 2016, to the end of February 2017. This center is a privet individual center that offers a wide range of dietary services like, weight loss, medical nutrition, eating disorders, sports nutrition, and others. All participants were new cases and were evaluated at the first visit. They were included if had 20–60 years old and satisfied to participate in the study. The exclusion criteria were alcohol consumption (> 40 g/day for males, > 20 g/day for females), diabetes mellitus, history of chronic diseases that impact metabolic condition like cancer, renal failure, and thyroid gland dysfunction according to self-report of physician-diagnosed and all those who for any reason followed a special diet in last 3 months.

### Methods

All methods were carried out in accordance with relevant guidelines and regulations such as Declaration of Helsinki guidelines and was approved by the Institutional Review Board (IRB) of Fasa University of Medical Sciences (Code: IR.FUMS.REC.1396.230). Written informed consent was obtained from all the participants and/or their legal guardian(s).

The age (years), gender, and complete medical histories were recorded as a self-completed questionnaire under the supervision of a trained nurse. Weight (closest 0.01 kg) and height (closest 0.1 cm) were measured using a stadiometer and digital scale (InBody 770, InBody Co., Ltd. South Korea) on the standing position and wearing light clothes without shoes.

A semi-quantitative food frequency questionnaire (FFQ), which contained 168 food items, was used to gather the dietary intake over the past year. The questionnaire’s validity and reliability have been approved previously^55^. In the FFQ, each person expressed the frequency of consuming each item with respect to the standard amount in the past year. The specified values of each food item were converted into grams per day using a manual for household measures. For best results, some similar food from 168 food elements were merged and categorized into 40 major food groups, like similar studies on the Iranian population such as Rezazadeh^26^ and Zareei^56^.

Transient elastography was performed with FibroScan (Echosens, Paris, France)^57^ using the standard probe (also named the M probe) by an individual sonographer. The fat in the liver was measured by the appropriate Controlled Attenuation Parameter (CAP) with the decibels per meter (dB/m) units^58^. The CAP scores lower than 238 dB/m indicate a S0 steatosis grade with 0–11% extent of the fat in the liver and was defined as a normal liver. In that, 238–260 dB/m was defined as mild fatty liver with S1 steatosis grade and 11 to 33% extent of the fat in the liver, 260–293 dB/m was defined as moderate fatty liver with S2 steatosis grade, and 33–66% extent of fat in the liver, and the CPA scores more than 293 dB/m indicate S3 steatosis grade and above 66% extent of the fat in the liver, it was defined as severe fatty liver.

### Network construction

Forty food groups extracted from FFQ were considered as the nodes of the CCN. The data set was 100 times randomly split into two sets, the test and the validation. Spearman correlation coefficient between each pair of food groups was calculated in the test and validation set separately. If this correlation was significant in both test and validation sets in all 100 repeats and the average of correlation coefficients of 100 test sets was greater than 0.2, then two food groups were considered as connected, otherwise disconnected (supplementary section II). The data set of food group consumption was binarized for each participant as 0, not consumed, and 1, consumed according to quartiles of consuming data (supplementary section III). The sum of the binarized consumption was regarded as the weight of the network’s nodes and the correlation coefficient as the weight of its edges.

### Differential co-consumption network

Two distinct networks were formed using the above method and thresholds for males and females separately. The differential co-consumption network was reconstructed using the same nodes (food groups) and edges which were weighted 1 (connected), provided that they appeared only in one of the male or female networks (supplementary section IV).

### Age trend network

We categorized participants' age into three categories, age under 30 (< 30) (n = 347), age between 30 and 50 (n = 751), and age over 50 (> 50) (n = 402). To study each group in detail, three separate networks were constructed using the above method and thresholds. The age trend co-consumption network was reconstructed using the same nodes (food groups) and edges which were weighted 1 (connected) if they had an increasing correlation. We defined the increasing correlation with one of two conditions. First, the correlation coefficient between each two food groups had an increase in the older group network in comparison to the younger group network. Second, the correlation coefficient in the > 50 network had more than 0.25 increase compared to the < 30 network (supplementary section V).

### Community detection

We used the label propagation algorithm for community detection in network^59^. In this algorithm, the labels were randomly assigned to nodes. Then the node labels were replaced with the neighbor's frequently appeared labels. The algorithm was applied until there were not any labels to be replaced. Since this algorithm may yield dissimilar answers, about 50 different initial values were tested and the most frequent answer was selected as the finalized partitioned network. The modularity score was calculated by Clauset and Newman^60^.

### Module membership detection

We used the hypergeometric distribution to categorize each participant into healthy and unhealthy clusters^61^. A probability table was obtained by employing the hypergeometric distribution, along with a probability lower than 0.1 was chosen as the criteria for the healthy or unhealthy module membership (supplementary section VI). According to the probability threshold, participants were assigned three labels as the members of the healthy or unhealthy modules and others. The last group was made up of participants that were members of either both modules or none of them. Then, a comparison was made between them in terms of age, energy intake, BMI, and liver steatosis index (CAP score), via the independent sample t-test.

### Module-related cliques

First, we identified the main CCN cliques and counted the number of healthy and unhealthy members who had consumed all the food groups, for each clique. Then, the chi-square test was used to compare the proportion of consumers in both groups. Next, the *P*-value was adjusted by the false discovery rate (FDR) correction^62^. And finally, a *p*-value less than 0.05 determined the module-related clique.

### Diseases differential co-consumption network

The participants were classified into two separate populations with a CAP score < 238 dB/m (a normal liver) and ≥ 293 dB/m (hepatic steatosis). We constructed two separate networks, using the differential co-consumption network method and threshold. The differential co-consumption network was reconstructed by the same nodes (food groups) and edges which were weighted 1 (connected) as long as they appeared only in one of the healthy or NAFLD networks.

### Software

The igraph an R package, was used to generate networks^63^ and all the computations were done in the “R” statistical package (R Core Team Version 3.5.0 2018)^64^ and Microsoft Excel (Microsoft Co., Redmond, WA).

### Ethics approval and consent to participate

The Ethics Committee of Fasa University of Medical Sciences approved the research protocol with No. IR.FUMS.REC.1396.230.

### Supplementary Information

Supplementary Information.

## Data Availability

The datasets used and/or analysed during the current study available from the corresponding author.

## Abbreviations

BMI: Body mass index
CAP: Controlled attenuation parameter
CCN: Co-consumption network
FDR: False discovery rate
FFQ: Food frequency questionnaire
FLI: Fatty liver index
LASSO: Least absolute shrinkage and selection operator
NAFLD: Non-alcoholic fatty liver disease
SD: Standard deviation
SUN: Seguimiento universidad de Navarra
