# Supplementary material for: Food co-consumption network as a new approach to dietary pattern in non-alcoholic fatty liver disease
Source: Sci Rep. 2023 Nov 24;13:20703. doi: 10.1038/s41598-023-47752-y (PMC10673913; doi:10.1038/s41598-023-47752-y)
Supplement: Supplementary file 1 — Supplementary Information. [file 41598_2023_47752_MOESM1_ESM.docx]

**Supplementary**

**Food Co-consumption Network as a New Approach to Dietary Pattern in Non-Alcoholic Fatty Liver Disease**

**Mohammad Mehdi Naghizadeh, Saeed Osati**

**Reza Homayounfar, Ali Masoudi-Nejad**

**Results:**

**Section I**


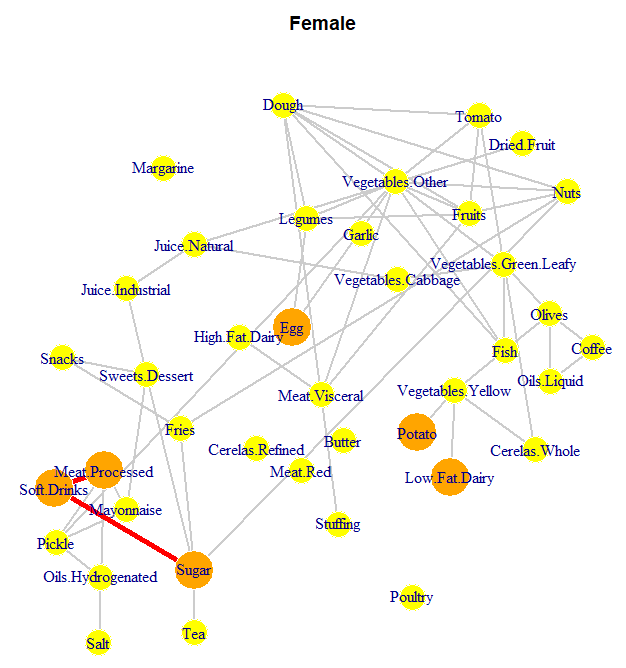

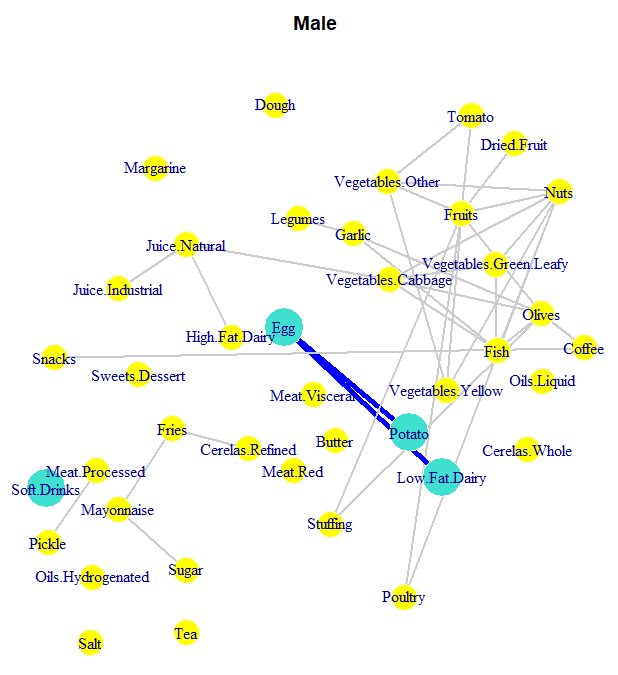


Figure S1: The Co-consumption network (CCN) for males (left) and females (right). The males were consuming soft drinks independently but were co-consuming egg with potato, and low-fat dairy. Vice versa, the females were consuming egg independently but were co-consuming soft drinks with processed meats and sugar.


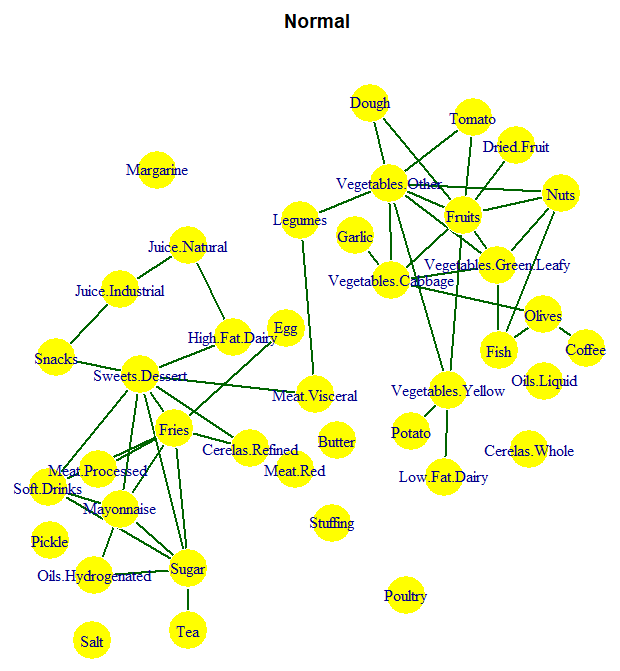

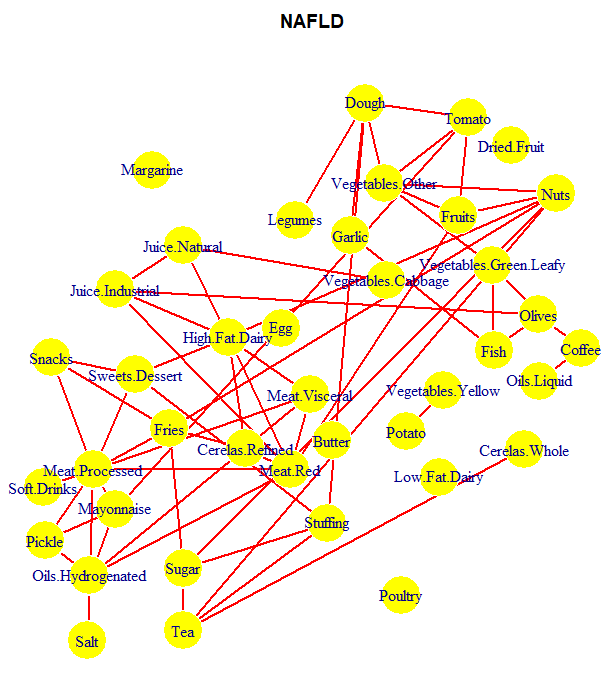


Figure S2: The Co-consumption network (CCN) for participant with normal liver (left) and NAFLD (right).

**Methods**

**Section II**


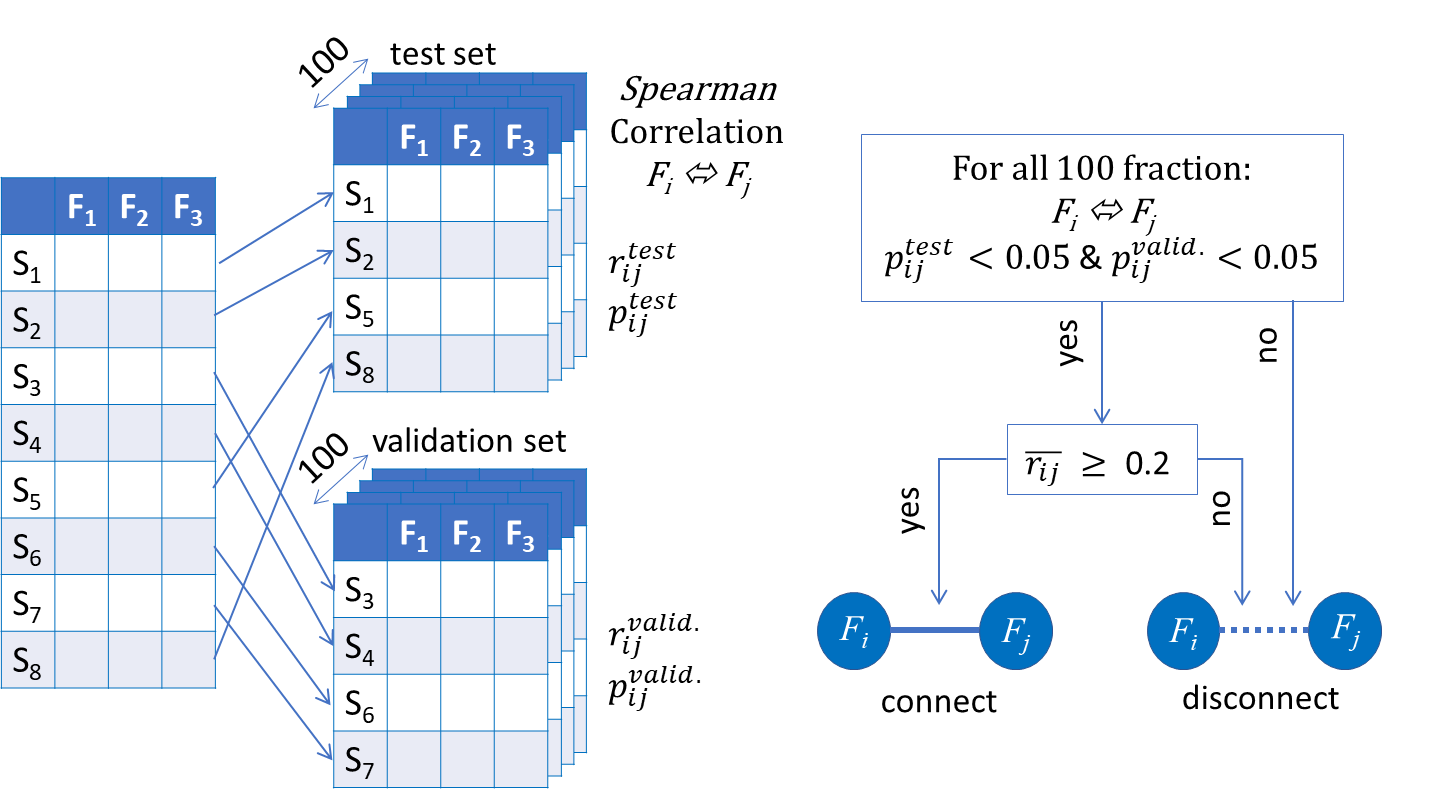
**Construction of Co-Consumption Network (CCN)**

Figure S3: determining of edges connection

Each network is defined by two sets, the nodes and the edges. The nodes in the CCN were forty food groups extracted from FFQ. For determining the edges, the food consumption dataset was split into two separate test and validation datasets. *Spearman*'s correlation coefficient of each food group like *F_i_* and *F_j_* was calculated as $r_{ij}^{test}$ in the test set and $r_{ij}^{valid.}$ in the validation set, also $p_{ij}^{test}$and $p_{ij}^{valid.}$were the p-values of them respectively. The non-parametric correlation coefficient was selected due to the frequency of food consumption groups had highly departure from the normal distribution. This process was repeated 100 times.

The food groups *F_i_* and *F_j_* were considered connected if they pass two criteria. The first, if the correlation was significant in the test set and related validation set in all 100 fractions. The second, if the average correlation coefficient of 100 test sets was greater a soft threshold ($\bar{r_{ij}}=\left( \frac{1}{100} \right)\sum_{1}^{100} r_{ij}^{test}\geq0.2$). According to the distribution of correlation coefficients in test sets ($r_{ij}^{test}$), 0.2 was chosen as the soft threshold (supplementary figure S4). Therefore, after passing the first criteria and if $\bar{r_{ij}}$ ≥ 0.2 then *F_i_* and *F_j_* were connected.

Figure S4: Distribution of average Spearman correlation coefficient between food groups. The correlation coefficients greater than 0.2 that were chosen as the connection were higher than the third quartile (Q3).

**Section III**


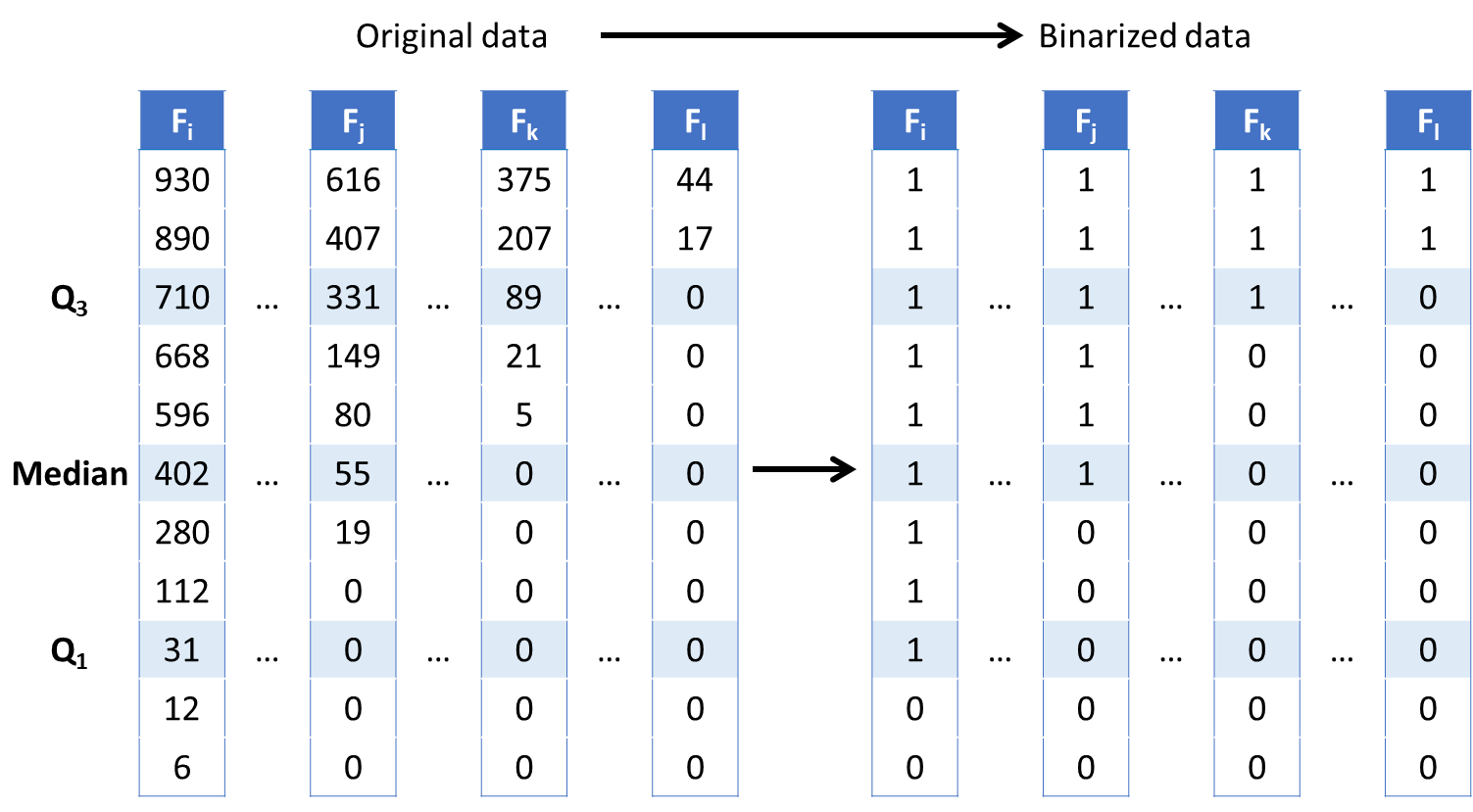
**Binarizing of food consumption data set.**

Figure S5: binarizing the data set

Since the consumption of the food groups showed a high standard deviation and had a zero-inflated distribution, the values of consumption were binarized by the cut-points. The consumption values which were greater than the cut-point were assigned 1 (consumed) and the others were given 0.

The first quartile was chosen as the cut-point if it was not zero (*F_i_* column in the figure). In this case, the greater consumption from the first quartile was considered as 1 and below it as 0. If the first quartile was zero but the median existed (*F_j_* column in the figure) then the median was chosen as the cut point. If the median was zero but the third quartile existed (*F_k_* column in the figure) then the upper quartile was chosen as the cut point. Any consumption of food groups was assigned 1 as long as the upper quartile was zero (*F_l_* column in the figure).

Section IV

Differential network


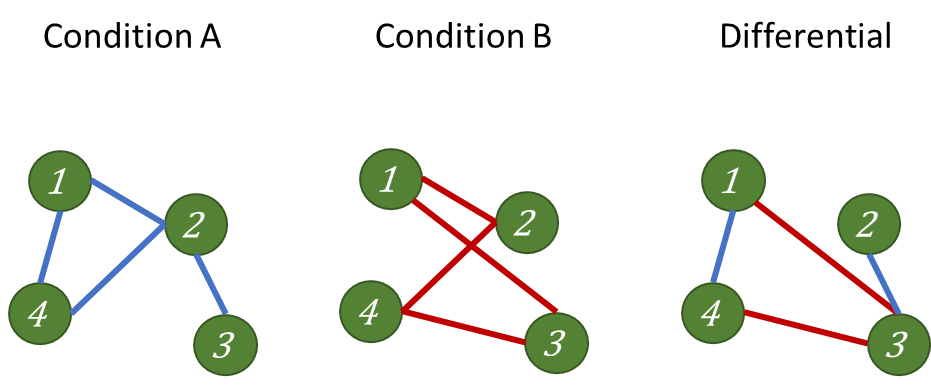


Figure S6- Differential network. At the last network the edges that appeared in only one condition A or B was presented.

Two distinct networks were formed for two different conditions separately. The differential network was reconstructed using the same nodes and edges that appeared only in one of the conditions networks.

**Section V**

**Age trend network**


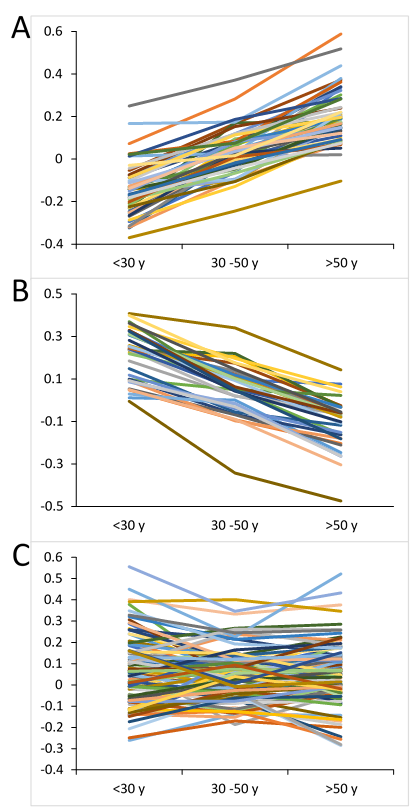
To construct the age trend network, the dataset was divided into three subsets according to age. (<30, 30 to 50, and >50 years). Then Spearman's correlation coefficient between food groups was calculated separately in every three subsets. For each food groups *F_i_* and *F_j_* three separate correlation coefficient were calculated ($r_{ij}^{<30}$, $r_{ij}^{30-50}$, $r_{ij}^{>50}$) that could have an increasing manner ($r_{ij}^{<30}$ < $r_{ij}^{30-50}$ < $r_{ij}^{>50}$, Figure S7.A) or decreasing manner ($r_{ij}^{<30}$ > $r_{ij}^{30-50}$ > $r_{ij}^{>50}$, Figure S7.B) or otherwise (Figure S7.C). The age trend network consisted of food groups as nodes set and increasing correlation as edges set. Increasing correlation was defined as firstly had increasing manner coefficients ($r_{ij}^{<30}$, $r_{ij}^{30-50}$, $r_{ij}^{>50}$), and second, the correlation coefficient in the last age group network had more than 0.25 increase compared to first age group network ($r_{ij}^{>50}- r_{ij}^{<30}>0.25$).

Figure S7: Trend of the correlation coefficient between food groups in the three age categories (<30, 30-50, >50). A. The increasing coefficients B. the decreasing coefficients was presented in the chart. C. The coefficients that had not increasing or decreasing manner.

**Section VI**

**Module membership detection**

The hypergeometric distribution was employed to put each participant in the healthy and unhealthy clusters. This distribution determined the probability of *x* success in *n* draws as long as *n* was a sample without the replacement of a population with size *N* that was partitioned into *K* success and *N-K* failures. In the present study, *N* was 40 food groups which were divided into unhealthy (*K* = 20) and healthy (*N-K* = 18) separate communities. Each participant could consume *n* food groups, *k* of them was unhealthy and *n-k* was healthy. So, the probability of *P* (*x* = *k*) was calculated using the following equation:

$$P_{X}\left( x=k \right)=\frac{\left( \begin{matrix} K \\ k \end{matrix} \right)\left( \begin{matrix} N-K \\ n-k \end{matrix} \right)}{\left( \begin{matrix} N \\ n \end{matrix} \right)}$$

Were $\left( \begin{matrix} Y \\ y \end{matrix} \right)= \frac{Y!}{y!\left( Y-y \right)!}$ Is y-combination of Y.

A probability table was obtained from employing the hypergeometric distribution, along with the above-mentioned parameters, and a P < 0.1 was chosen as criteria for one module membership. According to the probability threshold, participants were assigned three labels as the members of the healthy or unhealthy modules, and others. The last group was made up of participants that were the members of either both the modules or none of the modules. Then, a comparison was made between them in terms of age, the energy intake, the BMI, and the fatty liver index, respectively via the independent sample t-test.
